# Associations Between Metabolic Syndrome Index, Systemic Inflammatory Index, and Arthritis Subtype in the U.S. and Chinese Adults: A Mediation Analysis

**DOI:** 10.1155/mi/9114075

**Published:** 2026-09-02

**Authors:** Xiumin Jiang, Danting Wang, Zhuwei Zheng, Guangpu Zhu, Yuchao Zhang, Xianglong Feng, Huizhong Fu, Jiehui Fu, Shan Gao, Xiaowen Lian, Weiquan Zeng

**Affiliations:** ^1^ Fujian Rehabilitation Hospital, Fujian University of Traditional Chinese Medicine Subsidiary Rehabilitation Hospital, Fuzhou, China; ^2^ Fujian Key Laboratory of Rehabilitation Technology, Fuzhou, China; ^3^ Nursing College, Fujian University of Traditional Chinese Medicine, Fuzhou, China, fjtcm.edu.cn

**Keywords:** arthritis subtypes, mediation analysis, metabolic dysregulation, systemic inflammation index

## Abstract

**Background:**

Arthritis represents a spectrum of chronic inflammatory conditions, with growing evidence linking metabolic dysregulation to disease pathogenesis. While composite metabolic indices have been proposed as potential markers for arthritis, their associations with specific disease subtypes and applicability across diverse ethnic populations remain insufficiently characterized.

**Methods:**

This study analyzed data from 15,015 participants in the U.S. National Health and Nutrition Examination Survey (NHANES) and 3848 subjects from a Chinese hospital‐based cohort. Associations between triglyceride‐glucose (TyG) index/TyG–body mass index (TyG–BMI) and arthritis subtypes (osteoarthritis [OA], rheumatoid arthritis [RA], and gout) were assessed using multiple logistic regression and restricted cubic splines (RCS) with adjustment for age and gender. Mediation analysis evaluated the role of the systemic immune‐inflammation index (SII). Predictive performance was compared using area under the curve (AUC).

**Results:**

Elevated TyG and TyG–BMI were significantly associated with higher odds of multiple arthritis subtypes in both cohorts. In NHANES, the highest TyG quartile was linked to 26%–64% higher odds compared with the lowest quartile, while TyG–BMI showed stronger associations (ORs 2.25–2.76). Similarly, TyG–BMI was strongly associated with RA (OR = 4.77) and gout (OR = 5.46) in the Chinese cohort. Dose–response analyses confirmed significant nonlinear positive relationships between TyG/TyG–BMI levels and arthritis risk. Systemic inflammation mediated a substantial proportion of these associations (all *p* < 0.05). Predictive performance differed ethnically: TyG–BMI performed best in the U.S. cohort (AUCs 0.599–0.617), whereas the TyG index was superior in the Chinese cohort (AUCs 0.647–0.719).

**Conclusions:**

This cross‐population study demonstrates that TyG and TyG–BMI indices are positively and dose‐dependently associated with multiple arthritis subtypes, with systemic inflammation serving as a partial mediator. These findings underscore the utility of TyG and TyG–BMI indices in identifying at‐risk individuals for targeted early intervention and personalized management of arthritis.

## 1. Introduction

Arthritis constitutes a major source of chronic pain and disability globally, placing an escalating burden on healthcare systems worldwide [1, 2]. By 2030, an estimated 67 million adults in the United States alone are projected to receive a physician diagnosis of arthritis, representing nearly one‐quarter of the adult population [3]. Although traditionally classified into distinct etiopathogenic categories—such as mechanical degeneration, autoimmune dysregulation, and crystal deposition—growing evidence highlights metabolic dysregulation as a common pathogenic thread across various forms of arthritis [4, 5]. Notably, insulin resistance (IR), a core component of metabolic syndrome, has been implicated in promoting chronic low‐grade inflammation and disrupting articular homeostasis, suggesting its potential as a shared risk factor among different arthritis subtypes [6, 7]. Nevertheless, whether metabolic dysfunction contributes uniformly or differentially across arthritis types remains to be systematically elucidated. Furthermore, research has also explored natural therapeutic options for arthritis management. Plant‐derived compounds (*Filipendula ulmaria*) [8], combinatorial nutritional formulation [9], and flavonoids such as luteolin [10] have each shown anti‐inflammatory effects relevant to arthritic conditions. These findings complement the mechanistic perspective by highlighting inflammation as a shared therapeutic target.

The triglyceride‐glucose (TyG) index, calculated from fasting triglycerides and glucose, is widely recognized as a reliable and practical surrogate marker of IR [11]. More recently, the TyG–body mass index (TyG–BMI)—a product of the TyG index and BMI—has been proposed as an integrated metric that combines lipid‐glucose metabolism with overall adiposity, potentially offering superior predictive capacity for cardiometabolic diseases [12]. Given the close interplay between adipose tissue dysfunction, systemic inflammation, and IR, these indices may hold particular relevance in the pathogenesis of arthritis. Concurrently, chronic low‐grade inflammation serves as a hallmark of both metabolic dysregulation and many arthritic conditions [5, 13, 14]. The systemic immune‐inflammation index (SII), which incorporates neutrophil, lymphocyte, and platelet counts, has emerged as a comprehensive biomarker reflecting systemic inflammatory and immune status [15]. However, the extent to which SII explains the link between metabolic dysfunction and arthritis risk has not been formally evaluated.

To address these gaps, this study leverages two independent large‐scale cohorts: a nationally representative sample from the U.S. National Health and Nutrition Examination Survey (NHANES) and a hospital‐based cohort from China. We hypothesized that both the TyG index and TyG‑BMI are dose‑dependently associated with the risk of multiple arthritis subtypes, that systemic inflammation (SII) partially mediates these associations, and that the predictive performance of these indices may differ between the U.S. and Chinese populations. We aimed to elucidate the metabolic‑inflammatory pathways linking these indices to arthritis and to evaluate their potential as practical screening tools for risk stratification in diverse clinical settings through comprehensive analyses.

## 2. Methods

### 2.1. Study Design and Population

This cross‐sectional study utilized data from two independent cohorts: the U.S. NHANES and a hospital‐based cohort from China. The dual‐cohort design was employed to explore and validate the associations between metabolic indices and arthritis across diverse ethnic and clinical settings.

The U.S. population data were derived from seven consecutive 2‐year cycles (2005–2006 to 2017–2018) of the NHANES, a nationally representative survey employing a complex, stratified, multistage probability sampling design. All participants provided written informed consent, and the study protocol was approved by the National Center for Health Statistics Ethics Review Board. Initially, 70,190 participants no less than 20 years old were identified from seven consecutive NHANES cycles (2005–2006 to 2017–2018). We sequentially excluded individuals with missing diagnostic data for arthritis (*n* = 4000), those with missing laboratory data (*n* = 46,278), missing demographic data (*n* = 4897), yielding a final analytical sample of 15,015 adults. A detailed flowchart of the participant selection is provided in Figure S1.

To establish an Eastern population for comparative analysis, a hospital‐based cohort was retrospectively constructed from a tertiary center in China. Adults (aged ≥ 18 years) who underwent routine health examinations or were diagnosed with arthritis in the inpatient and outpatient departments between January 2022 and December 2025 were screened. Individuals with a history of malignancy, severe hepatic or renal dysfunction, acute infections, or missing essential data were excluded. This process yielded a final Chinese cohort of 3848 participants (Figure S2). The study was approved by the Institutional Review Board of Fujian University of Traditional Chinese Medicine Affiliated Rehabilitation Hospital (Approval No. 2025KY‐047‐01).

### 2.2. Assessment of Arthritis

In both cohorts, arthritis status was ascertained based on a physician diagnosis. In the NHANES cohort, participants were asked: “Has a doctor or other health professional ever told you that you had arthritis?” Those who answered “yes” were further queried: “Which type of arthritis was it?” Responses were categorized into osteoarthritis (OA), rheumatoid arthritis (RA), or other arthritis. For the Chinese cohort, arthritis diagnoses (including knee OA (KOA), RA, and gout) were extracted from electronic medical records based on standard diagnostic criteria documented by attending physicians.

### 2.3. Assessment of Metabolic Indices and Inflammatory Markers

The TyG index was calculated as: Ln [triglycerides (mg/dL) × fasting glucose (mg/dL)/2]. The TyG–BMI was derived by multiplying the TyG index by the BMI (kg/m^2^) [16]. The SII was computed from complete blood count parameters using the formula: SII = (platelet count × neutrophil count)/lymphocyte count, with all cell counts expressed as ×10^9^/L [17]. Laboratory methods for the NHANES are publicly available, and all assays in the Chinese cohort were performed in the hospital’s laboratory department, following standardized protocols.

### 2.4. Statistical Analysis

All statistical analyses were performed using R software (version 4.1.3). A two‐sided *p*‐value < 0.05 was considered statistically significant. For the NHANES cohort, continuous variables are presented as mean ± standard and categorical variables as percentages. For the Chinese cohort, standard descriptive statistics were applied, with continuous variables expressed as mean ± standard deviation as appropriate and categorical variables as frequencies (percentages). The associations of TyG and TyG‐–BMI (categorized into quartiles, Q1 as the reference) with arthritis subtypes were evaluated using multivariable logistic regression models. In the primary analysis, all models were adjusted for age and sex; unadjusted estimates were also computed. Results are expressed as odds ratios (ORs) with 95% confidence intervals (CIs).

Dose‐response relationships were examined using restricted cubic splines (RCS) with adjustment for age and gender [18]. Unadjusted analyses were also performed. The likelihood ratio test was used to assess the nonlinearity. Mediation analysis was conducted to evaluate the potential mediating role of SII in the relationship between metabolic indices (TyG and TyG–BMI) and arthritis subtypes. The direct and indirect effects were estimated using a quasi‐Bayesian Monte Carlo simulation approach with 5000 iterations [19]. Finally, the predictive performance of TyG, TyG–BMI, and SII for different arthritis subtypes was compared by calculating the area under the curve (AUC).

## 3. Result

### 3.1. Characteristics of Participants

The baseline characteristics of the included participants (15,015 from the NHANES and 3848 from the Chinese hospital‐based cohort) are presented in Table 1. Participants from the U.S. cohort were, on average, younger than those from the Chinese cohort (mean age 48.67 vs. 59.96 years). The proportion of male participants was 48.3% in the NHANES cohort and 44.66% in the Chinese cohort. Furthermore, mean values for BMI, TyG–BMI, and SII were higher in the U.S. cohort compared to the Chinese cohort (28.96 vs. 24.75; 249.56 vs. 219.86; and 524.71 vs. 463.72, respectively).

**Table 1:** Basic characteristics of participants from the two cohorts.

Characteristic	U.S. NHANES					Chinese hospital‐based cohort				
	All participants (*N* = 15,015)	Arthritis			Non‐arthritis (*N* = 11,956)	All participants (*N* = 3848)	Arthritis			Non‐arthritis (*N* = 1997)
		OA (*N* = 1102)	RA (*N* = 1503)	Other arthritis (*N* = 544)			KOA (*N* = 1438)	RA (*N* = 166)	Gout (*N* = 247)	
Age (year)	48.67 (17.70)	63.20 (13.02)	62.39 (13.41)	57.67 (14.21)	45.19 (17.02)	59.96 (13.06)	68.92 (11.14)	54.86 (11.28)	60.61 (13.26)	53.85 (10.48)
Male (%)	48.3	39.9	36.6	45.0	50.7	44.66	28.9	51.2	87.8	50.0
White *n* (%)	42.0	54.5	54.7	55.9	38.6	NA	NA	NA	NA	NA
BMI	28.96 (6.97)	30.57 (7.50)	31.28 (8.04)	30.99 (8.63)	28.43 (6.57)	24.75 (3.54)	25.53 (3.91)	25.76 (3.66)	26.04 (3.52)	23.95 (3.03)
Fasting blood glucose (mg/dL)	108.09 (34.03)	113.38 (34.84)	115.31 (38.47)	115.38 (41.36)	106.36 (32.76)	107.07 (31.53)	113.81 (39.84)	121.20 (48.94)	113.14 (33.24)	100.29 (18.42)
Triglyceride (mmol/L)	1.32 (0.75)	1.40 (0.72)	1.42 (0.75)	1.45 (0.80)	1.30 (0.75)	1.75 (1.18)	1.73 (0.94)	3.39 (2.66)	2.23 (2.12)	139.78 (1.58)
Total cholesterol (mmol/L)	4.95 (1.06)	4.99 (1.08)	4.98 (1.09)	4.95 (1.09)	4.95 (1.05)	5.25 (1.12)	5.01 (1.13)	5.55 (1.25)	4.93 (1.04)	5.43 (1.07)
White blood cell count (10^9^/L)	6.82 (2.43)	6.84 (2.30)	7.12 (2.73)	7.12 (2.08)	6.77 (2.41)	6.04 (1.90)	6.23 (1.95)	6.68 (1.87)	8.26 (2.35)	5.58 (1.55)
Neutrophil count (10^9^/L)	3.99 (1.76)	4.08 (1.67)	4.23 (1.81)	4.21 (1.70)	3.94 (1.76)	3.45 (1.59)	3.68 (1.77)	3.83 (1.30)	5.47 (2.11)	3.00 (1.10)
Lymphocyte count (10^9^/L)	2.05 (1.27)	1.97 (1.34)	2.05 (1.64)	2.07 (0.79)	2.06 (1.22)	1.93 (0.69)	1.88 (0.58)	2.11 (0.67)	2.20 (1.29)	1.92 (0.65)
Platelet count (10^9^/L)	244.46 (66.28)	241.72 (71.06)	240.78 (66.24)	247.36 (66.61)	245.05 (65.79)	234.96 (60.42)	231.52 (61.53)	247.73 (56.58)	258.01 (71.21)	233.53 (57.72)
Low‐density lipoprotein cholesterol (mmol/L)	2.94 (0.92)	2.90 (0.93)	2.89 (0.96)	2.88 (0.97)	2.95 (0.91)	3.25 (0.94)	3.10 (0.96)	3.25 (0.85)	2.96 (0.83)	3.38 (0.92)
High‐density lipoprotein cholesterol (mmol/L)	1.41 (0.42)	1.44 (0.41)	1.44 (0.46)	1.40 (0.42)	1.40 (0.41)	1.26 (0.33)	1.20 (0.31)	1.07 (0.26)	0.95 (0.25)	1.36 (0.31)
TyG	8.58 (0.63)	8.70 (0.57)	8.72 (0.63)	8.73 (0.64)	8.54 (0.63)	8.86 (0.56)	8.94 (0.48)	9.45 (0.90)	9.13 (0.57)	8.72 (0.53)
TyG–BMI	249.56 (67.30)	266.83 (71.13)	273.83 (77.22)	271.87 (82.71)	243.90 (63.56)	219.86 (37.66)	288.36 (38.34)	244.68 (48.33)	237.88 (37.42)	209.44 (32.51)
SII	524.71 (395.29)	567.31 (383.05)	578.10 (422.02)	555.28 (340.72)	512.69 (394.39)	463.72 (397.50)	511.33 (519.74)	460.60 (152.75)	812.16 (619.75)	386.61 (195.41)

Abbreviations: BMI, body mass index; KOA, knee osteoarthritis; OA, osteoarthritis; RA, rheumatoid arthritis; SII, systemic immune‐inflammation index; TyG, triglyceride‐glucose index; TyG–BMI, TyG–body mass index.

### 3.2. Association of TyG and TyG–BMI With Different Arthritis

**Figure 1:**
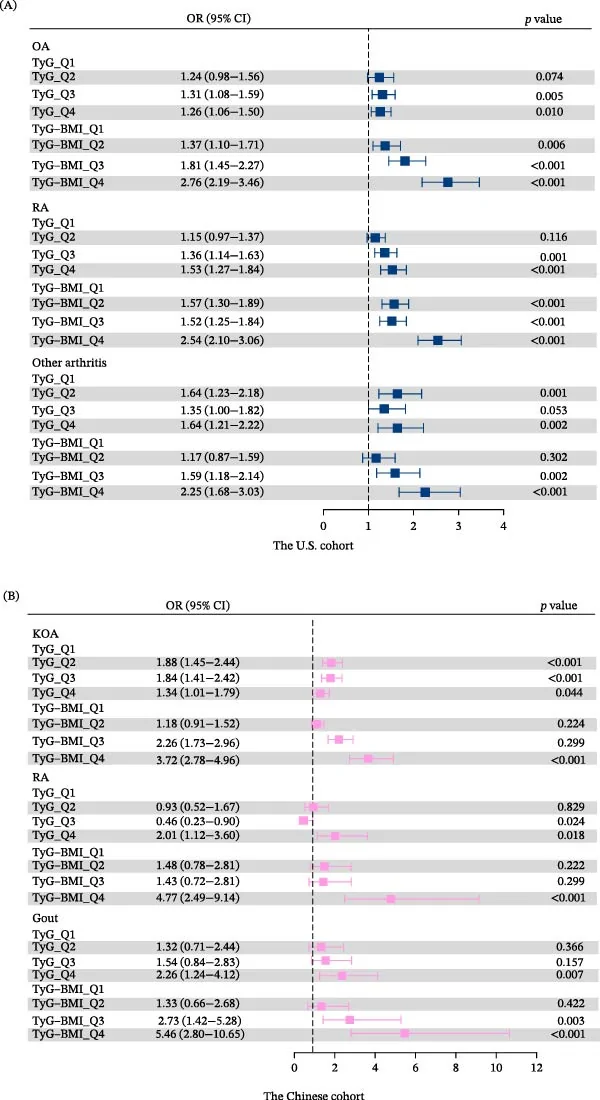
Association of TyG and TyG–BMI with different arthritis in the two cohorts. (A) The U.S. cohort; (B) The Chinese cohort. Adjusted for age and gender. KOA, knee osteoarthritis; OA, osteoarthritis; OR, odds ratio; RA, rheumatoid arthritis.

Multiple logistic regression analyses were performed to examine the associations of the TyG and TyG–BMI indices with various arthritis subtypes. As shown in Figure 1A, within the U.S. cohort, the highest quartile (Q4) of the TyG index was associated with significantly higher odds of OA, RA, and other arthritis types compared to the lowest quartile (Q1), with increases of 26%, 53%, and 64%, respectively. Furthermore, a higher TyG–BMI index was associated with higher odds for OA (2.76, 2.19–3.46), RA (2.54, 2.10–3.06), and other arthritis (2.25, 1.68–3.03). Similarly, in the Chinese cohort, the TyG index at Q4 was associated with higher odds of KOA (34%), RA (101%), and gout (126%) compared with Q1. The TyG–BMI index also showed significantly higher odds for KOA (3.72, 2.78–4.96), RA (4.77, 2.49–9.14), and gout (5.46, 2.80–10.65) (Figure 1B). The results shown in Figure 1 were adjusted for age and sex, while the corresponding unadjusted estimates are available in Table S1 (the U.S. cohort) and Table S2 (the Chinese cohort). Collectively, these results consistently indicated a positive association between elevated metabolic indices and higher odds for multiple arthritis subtypes across both cohorts.

### 3.3. Dose–Response Relationships

**Figure 2:**
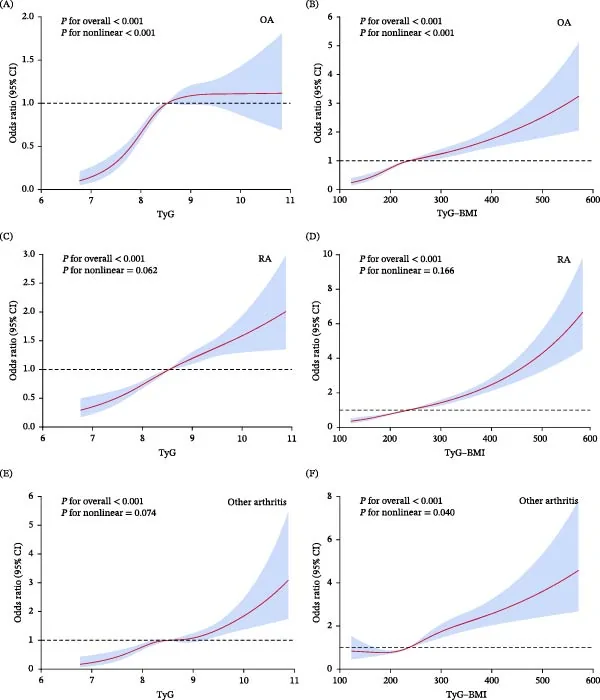
Generalized additive models illustrating the association of TyG and TyG‐BMI with arthritis subtypes in the U.S. cohort. (A) TyG for OA, (B) TyG‐BMI for OA, (C) TyG for RA, (D) TyG‐BMI for RA, (E) TyG for other arthritis, and (F) TyG‐BMI for other arthritis. Adjusted for age and gender.

**Figure 3:**
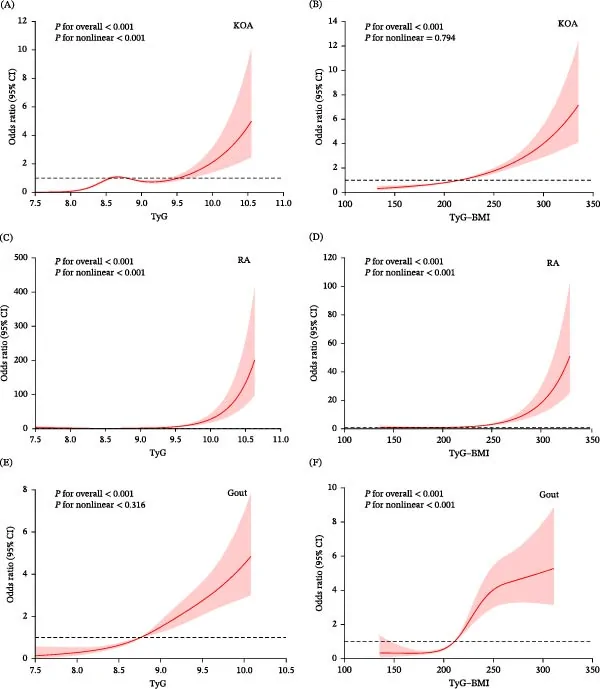
Generalized additive models illustrating the association of TyG and TyG‐BMI with arthritis subtypes in Chinese hospital‐based cohort. (A) TyG for KOA, (B) TyG‐BMI for KOA, (C) TyG for RA, (D) TyG‐BMI for RA, (E) TyG for gout, and (F) TyG‐BMI for gout. Adjusted for age and gender.

Dose–response curve analysis using the RCS revealed the nonlinear association of TyG and TyG–BMI with different arthritis subtypes. As depicted in Figure 2 (with adjustment for age and sex), within the U.S. cohort, the TyG index showed a positive correlation with OA (*P*‐nonlinear < 0.001). Furthermore, TyG–BMI exhibited a significant positive association with OA (*P*‐nonlinear < 0.001) and other arthritis (*P*‐nonlinear = 0.040). In the Chinese cohort (Figure 3, also adjusted for age and sex), the TyG index was positively correlated with KOA and RA (all *P*‐nonlinear < 0.001), while TyG–BMI showed a positive correlation with RA and gout (all *P*‐nonlinear < 0.001). It should be noted that CIs for the RCS estimates widened at the tails of the TyG and TyG–BMI distributions, particularly in the Chinese cohort, due to sparse observations at these extreme levels. Nevertheless, overall nonlinear trends remained stable across the central exposure range where most participants were clustered, and likelihood ratio tests for overall associations were highly significant. Thus, while extreme estimates warrant cautious interpretation, the dose–response patterns are considered robust within the observed data range. Unadjusted results are provided in Figure S3 (the U.S. cohort) and Figure S4 (the Chinese cohort).

### 3.4. Mediating Role of Inflammatory Markers

**Figure 4:**
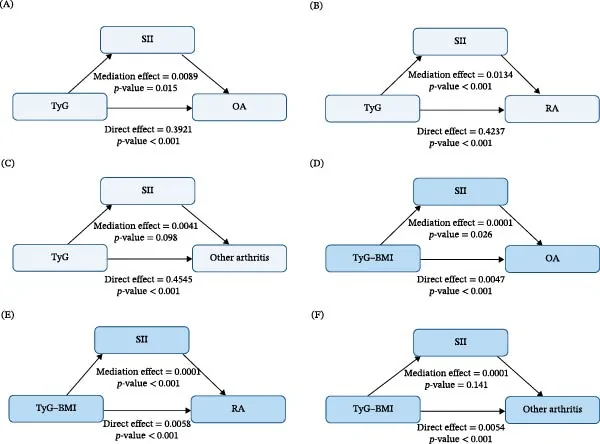
Mediation analysis showing the proportion of the association between TyG/TyG‐BMI and arthritis subtypes accounted for by systemic immune‐inflammation index (SII) in the U.S. cohort. (A) TyG and OA; (B) TyG and RA; (C) TyG and other arthritis; (D) TyG‐BMI and OA; (E) TyG‐BMI and RA; (F) TyG‐BMI and other arthritis. Adjusted for age and gender.

**Figure 5:**
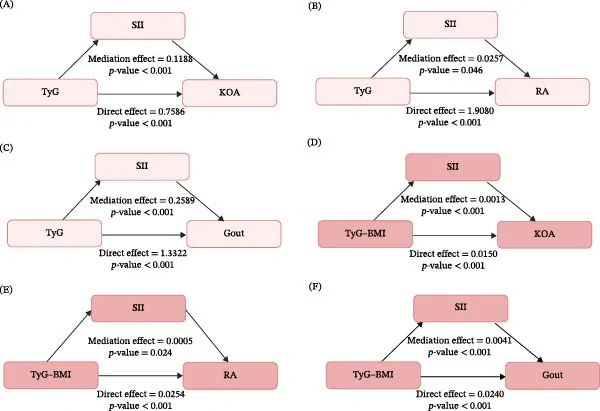
Mediation analysis showing the proportion of the association between TyG/TyG‐BMI and arthritis subtypes accounted for by systemic immune‐inflammation index in Chinese hospital‐based cohort. (A) TyG and KOA; (B) TyG and RA; (C) TyG and gout; (D) TyG‐BMI and KOA; (E) TyG‐BMI and RA; (F) TyG‐BMI and gout. Adjusted for age and gender.

Mediation analyses were conducted to investigate the role of inflammatory markers in the association between metabolic indices and arthritis. As shown in Figure 4, within the U.S. cohort, SII explained a significant proportion of the positive statistical association on the relationship of both TyG and TyG–BMI with OA and RA (all *p* < 0.05). Similarly, in the Chinese cohort, SII also accounted for a significant portion of the association in the relationships of TyG and TyG–BMI with KOA, RA, and gout (all *p* < 0.05; Figure 5). The estimates presented in Figures 4 and 5 were adjusted for age and sex; the corresponding unadjusted results are available in Figures S5 (the U.S. cohort) and Figures S6 (the Chinese cohort). Collectively, these results indicated that systemic inflammation, measured by SII, partially explained the connection between metabolic dysregulation and the arthritis subtypes.

### 3.5. Predictive Performance of Metabolic Indices for Arthritis

**Figure 6:**
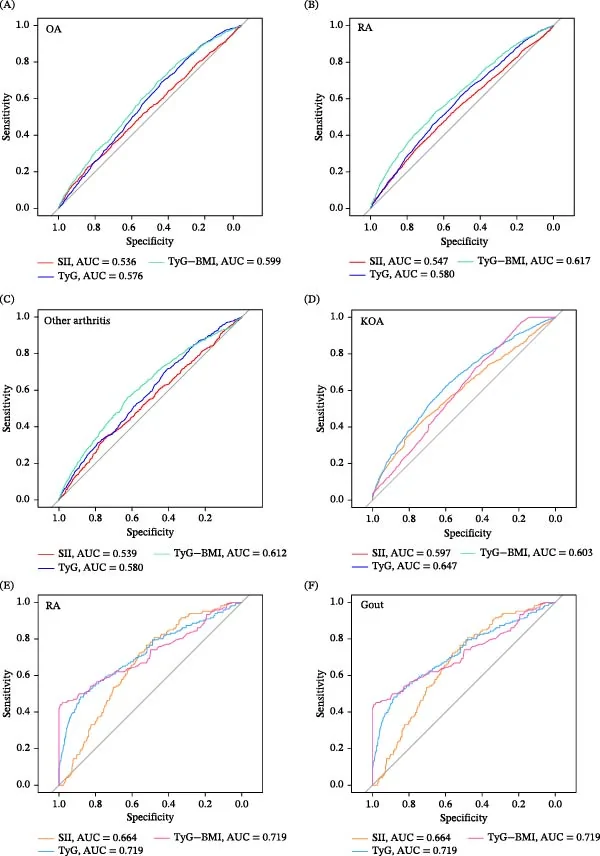
Receiver operating characteristic curve and area under the curve analysis for predicting arthritis subtypes in the two cohorts. (A–C) OA, RA, and other arthritis in the U.S. cohort; (D–F) KOA, RA, and gout in the Chinese cohort.

The predictive performance of TyG, TyG–BMI, and SII for different arthritis subtypes was compared by the AUC (Figure 6). In the U.S. cohort, TyG–BMI demonstrated the highest predictive ability for OA, RA, and other arthritis, with AUC value of 0.599, 0.617, and 0.612, respectively, followed by the TyG index (AUCs: 0.576, 0.580, and 0.580). Conversely, in the Chinese cohort, the TyG index showed the strongest predictive advantage for KOA, RA, and gout, with corresponding AUCs of 0.647, 0.719, and 0.719, respectively, outperforming TyG–BMI (AUCs: 0.603, 0.719, and 0.719). Overall, these analyses highlighted TyG–BMI as a superior predictor in the Western population, whereas the TyG index appeared more relevant for arthritis prediction in the Eastern cohort, underscoring potential ethnic or population‐specific utility of these metabolic markers.

## 4. Discussion

This study comprehensively analyzed arthritis subtypes across two ethnically and clinically distinct populations. By integrating novel metabolic indices with an established inflammatory marker, our findings provide multi‐dimensional evidence that metabolic dysfunction, particularly IR and adiposity, is robustly associated with arthritis risk and that this relationship is partially mediated by systemic inflammation.

This study found that higher TyG and TyG–BMI indices were significantly associated with greater odds of various arthritis types, thereby extending and strengthening the accumulating evidence connecting metabolic dysregulation with musculoskeletal disorders [20, 21]. The TyG index, a well‐established surrogate marker of IR, has been linked to the risk of OA, potentially mediated by mechanisms such as chronic low‐grade inflammation, oxidative stress, and dysregulated chondrocyte metabolism [20, 22, 23]. The even stronger associations observed for TyG–BMI, particularly in the U.S. cohort, suggested that the confluence of IR and increased adiposity exerts a synergistic detrimental effect. Adipose tissue is an active endocrine organ secreting pro‐inflammatory adipokines (e.g., leptin and resistin) and cytokines that could perpetuate joint inflammation and degradation, providing a plausible biological pathway for our observations [24]. The consistency of these associations across two ethnically and geographically distinct cohorts strengthens the argument for a fundamental metabolic component in arthritis etiology beyond traditional risk factors. Notably, the ORs for TyG–BMI in the Chinese cohort, particularly for RA (OR = 4.77) and gout (OR = 5.46), were substantially higher than those for the TyG index. This numerical disparity is partly attributable to the multiplicative structure of the TyG–BMI, which yields a broader numerical scale than TyG alone. To exclude the possibility that these high ORs arose from multicollinearity, we assessed variance inflation factors (VIFs) in the regression models; all VIFs were below 2, confirming that collinearity did not unduly inflate the estimates. Therefore, the observed stronger associations for TyG–BMI more plausibly reflect a synergistic effect of combined IR and adiposity rather than a statistical artifact.

Our mediation analysis offers mechanistic insight by identifying SII—a composite marker reflecting systemic immune‐inflammatory balance—as a significant partial mediator. This was especially consistent for the TyG–BMI index in both cohorts. IR and visceral adiposity are known to drive a state of meta‐inflammation, characterized by activation of innate immune pathways (e.g., NLRP3 inflammasome) and elevated circulating levels of interleukin‐1β, interleukin‐6, and tumor necrosis factor‐alpha [25, 26]. These inflammatory mediators are pivotal in the synovitis and cartilage breakdown seen in RA and OA [27, 28]. Therefore, our results posited that metabolic abnormalities may contribute to arthritis risk, at least in part, by fueling a pro‐inflammatory systemic environment.

An intriguing and potentially clinically relevant finding is the divergent predictive performance of the metabolic indices between the two cohorts. In the U.S. NHANES cohort, the TyG–BMI demonstrated superior predictive ability for arthritis subtypes, whereas in the Chinese cohort, the TyG index alone showed the strongest predictive value. This discrepancy may be attributed to several inter‐population differences. First, the mean BMI in the U.S. cohort was notably higher (28.96 vs. 24.75 kg/m^2^), placing it in the overweight range. In a population with prevalent adiposity, a combined index integrating IR and BMI may capture a more comprehensive metabolic risk profile [29]. Conversely, in the Chinese cohort with a lower average BMI, IR, possibly driven by factors like dietary patterns or genetic predisposition, might be the more dominant driver of arthritis risk, diminishing the added value of BMI in the index [30]. This aligns with the notion that the TyG index can identify IR even in nonobese individuals [31]. Second, ethnic variations in body composition, fat distribution (e.g., higher visceral adiposity at lower BMIs in Asian populations), and genetic susceptibility to metabolic‐inflammatory crosstalk could contribute to these differences [32, 33]. This highlights the importance of context and population characteristics when selecting metabolic screening tools for risk stratification.

Several limitations should be acknowledged. First, the cross‑sectional design precludes causal inference, and reverse causation is plausible. Second, residual confounding from unmeasured factors (e.g., medication use, physical activity, dietary quality, and genetic susceptibility) may persist. Third, the substantial reduction in the NHANES sample due to missing data may introduce selection bias; the hospital‑based Chinese cohort may overrepresent severe cases with enriched comorbidities, and diagnostic reliance on self‑report or clinical records without standardized imaging may introduce heterogeneity in outcome definitions—both of which limit direct comparability with NHANES. Fourth, cross‑population comparisons are further complicated by temporal mismatches (NHANES 2005–2018 vs. Chinese 2022–2025), which may introduce unadjusted period effects. Fifth, mediation analysis was performed on contemporaneously measured variables; thus, indirect effects reflect statistical associations rather than causal pathways. Finally, the predictive performance of the indices was modest (AUCs 0.599–0.719), supporting their role as screening tools rather than diagnostic markers. These findings warrant validation in prospective population‑based cohorts across diverse ethnic groups.

## 5. Conclusion

This study provided cross‐population evidence that metabolic indices—particularly TyG and TyG–BMI—are significantly associated with the risk of multiple arthritis subtypes and that systemic inflammation partially mediates this relationship. Our findings highlighted the potential utility of simple, low‐cost screening tools for identifying high‐risk individuals in diverse clinical and public health settings.

## Author Contributions

**Xiumin Jiang:** conceptualization, methodology, formal analysis, software, visualization, writing – original draft, writing – review and editing. **Danting Wang:** data curation, methodology. **Zhuwei Zheng:** data curation, software. **Guangpu Zhu:** data curation, software. **Yuchao Zhang:** data curation, visualization. **Xianglong Feng:** data curation, visualization. **Huizhong Fu:** data curation, supervision. **Jiehui Fu:** visualization, project administration. **Shan Gao:** visualization, project administration. **Xiaowen Lian:** project administration, supervision, writing – review and editing. **Weiquan Zeng:** conceptualization, funding acquisition, project administration, supervision, writing – review and editing.

## Funding

This research was funded by the National Science Foundation of China (Grant 82575107), Fujian Province Science and Technology Innovation Joint Fund Project (Grant 2024Y9523), Traditional Chinese Orthopedics Open subject of FJTCM (Grant XGS2023002), High‐Level Key Discipline Construction Project in TCM Rehabilitation under the National Administration of Traditional Chinese Medicine (Grant zyyzdxk‐2023102), NATCM’s Project of High‐level Construction of Key TCM Disciplines (Traditional Chinese Orthopedics) (Grant zyyzdxk‐2023106), and Fujian Rehabilitation Industry Research Institute Technology Innovation Platform Construction Project (Grant 2015Y2001).

## Ethics Statement

This study was conducted in accordance with the Declaration of Helsinki. For the U.S. cohort, publicly available, de‐identified data from the National Health and Nutrition Examination Survey (NHANES) were used, with prior approval by the NCHS Research Ethics Review Board and documented participant consent. For the Chinese hospital‐based cohort, the study protocol was approved by the Institutional Review Board of Fujian University of Traditional Chinese Medicine Affiliated Rehabilitation Hospital. All data were anonymized throughout the study to ensure participant confidentiality.

## Conflicts of Interest

The authors declare no conflicts of interest.

## Supporting Information

Additional supporting information can be found online in the Supporting Information section.

## Supporting information


**Supporting Information** Figure S1: Flowchart of participants selection in NHANES cohort. Figure S2: Flowchart of participants selection in hospital‐based cohort. Figure S3: Generalized additive models illustrating the association of TyG and TyG–BMI with arthritis subtypes in the U.S. cohort. (A) TyG for OA, (B) TyG‐BMI for OA, (C) TyG for RA, (D) TyG‐BMI for RA, (E) TyG for other arthritis, and (F) TyG‐BMI for other arthritis. Unadjusted for covariate. Figure S4: Generalized additive models illustrating the association of TyG and TyG–BMI with arthritis subtypes in Chinese cohort. (A) TyG for KOA, (B) TyG‐BMI for KOA, (C) TyG for RA, (D) TyG‐BMI for RA, (E) TyG for gout, and (F) TyG‐BMI for gout. Unadjusted for covariate. Figure S5: Mediation analysis showing the proportion of the association between TyG/TyG–BMI and arthritis subtypes accounted for by systemic immune‐inflammation index in the U.S. cohort. (A) TyG and OA; (B) TyG and RA; (C) TyG and other arthritis; (D) TyG‐BMI and OA; (E) TyG‐BMI and RA; (F) TyG‐BMI and other arthritis. Unadjusted for covariate. Figure S6: Mediation analysis showing the proportion of the association between TyG/TyG–BMI and arthritis subtypes accounted for by systemic immune‐inflammation index in Chinese hospital‐based cohort. (A) TyG and KOA; (B) TyG and RA; (C) TyG and gout; (D) TyG‐BMI and KOA; (E) TyG‐BMI and RA; (F) TyG‐BMI and gout. Unadjusted for covariates. Table S1: Associations of TyG index and TyG–BMI with arthritis subtypes in the U.S. NHANES cohort, unadjusted for covariates. Table S2: Associations of TyG index and TyG–BMI with arthritis subtypes in the Chinese hospital‐based cohort, unadjusted for covariates.

## Data Availability

Data will be made available upon request.
