# Supplementary material for: Associations Between Metabolic Syndrome Index, Systemic Inflammatory Index, and Arthritis Subtype in the U.S. and Chinese Adults: A Mediation Analysis
Source: Mediators Inflamm. 2026 Sep 2;2026:9114075. doi: 10.1155/mi/9114075 (PMC13539070; doi:10.1155/mi/9114075)
Supplement: Supplementary file 1 [file MI-2026-9114075-s001.docx]

**Supplementary materials**

**Supplementary Figure**

**Figure S1. Flowchart of participants selection in NHANES cohort.**

**Figure S2. Flowchart of participants selection in hospital-based cohort.**

**Figure S3.** Generalized additive models illustrating the association of TyG and TyG-BMI with arthritis subtypes in U.S. cohort, unadjusted for covariates.

**Figure S4.** Generalized additive models illustrating the association of TyG and TyG-BMI with arthritis subtypes in Chinese cohort, unadjusted for covariates.

**Figure S5**. Mediation analysis showing the proportion of the association between TyG/TyG-BMI and arthritis subtypes accounted for by systemic immune-inflammation index in the U.S. cohort, unadjusted for covariates.

**Figure S6.** Mediation analysis showing the proportion of the association between TyG/TyG-BMI and arthritis subtypes accounted for by systemic immune-inflammation index in Chinese hospital-based cohort, unadjusted for covariates.

**Supplementary Table**

**Table S1.** Associations of TyG index and TyG-BMI with arthritis subtypes in the U.S. NHANES cohort, unadjusted for covariates.

|  | **Odds Ratio** | **95%CI** | ***P* value** |
| --- | --- | --- | --- |
| **OA** |  |  |  |
| TyG_Q1 |  |  |  |
| TyG_Q2 | 1.49 | (1.21, 1.83) | 0.000 |
| TyG_Q3 | 1.63 | (1.32, 2.00) | 0.000 |
| TyG_Q4 | 1.50 | (1.20,1.86) | 0.000 |
| TyG-BMI_Q1 |  |  |  |
| TyG-BMI_Q2 | 1.43 | (1.15, 1.76) | 0.001 |
| TyG-BMI_Q3 | 1.72 | (1.39, 2.13) | 0.000 |
| TyG-BMI_Q4 | 2.12 | (1.70, 2.63) | 0.000 |
| **RA** |  |  |  |
| TyG_Q1 |  |  |  |
| TyG_Q2 | 1.07 | (0.89, 1.27) | 0.448 |
| TyG_Q3 | 1.22 | (1.02, 1.45) | 0.025 |
| TyG_Q4 | 1.30 | (1.08, 1.55) | 0.005 |
| TyG-BMI_Q1 |  |  |  |
| TyG-BMI_Q2 | 1.53 | (1.26, 1.83) | 0.000 |
| TyG-BMI_Q3 | 1.51 | (1.24, 1.83) | 0.000 |
| TyG-BMI_Q4 | 2.70 | (2.23, 3.25) | 0.000 |
| **Other arthritis** |  |  |  |
| TyG_Q1 |  |  |  |
| TyG_Q2 | 1.59 | (1.19, 2.11) | 0.002 |
| TyG_Q3 | 1.28 | (0.94, 1.73) | 0.107 |
| TyG_Q4 | 1.51 | (1.11, 2.05) | 0.007 |
| TyG-BMI_Q1 |  |  |  |
| TyG-BMI_Q2 | 1.16 | (0.85, 1.56) | 0.348 |
| TyG-BMI_Q3 | 1.59 | (1.17, 2.13) | 0.003 |
| TyG-BMI_Q4 | 2.32 | (1.72, 3.11) | 0.000 |
| Abbreviation: OA, osteoarthritis; RA, rheumatoid arthritis; BMI, body mass index; TyG, triglyceride-glucose index; TyG-BMI, TyG-body mass index; Q1-Q4, quartiles 1 to 4 ,with Q1 as the reference group. | | | |

**Table S2.** Associations of TyG index and TyG-BMI with arthritis subtypes in the Chinese hospital-based cohort, unadjusted for covariates.

|  | **Odds Ratio** | **95%CI** | ***P* value** |
| --- | --- | --- | --- |
| **KOA** |  |  |  |
| TyG_Q1 |  |  |  |
| TyG_Q2 | 1.79 | (1.44, 2.20) | 0.000 |
| TyG_Q3 | 1.64 | (1.31, 2.05) | 0.000 |
| TyG_Q4 | 1.25 | (0.99, 1.58) | 0.061 |
| TyG-BMI_Q1 |  |  |  |
| TyG-BMI_Q2 | 1.30 | (1.05, 1.61) | 0.015 |
| TyG-BMI_Q3 | 2.07 | (1.66, 2.57) | 0.000 |
| TyG-BMI_Q4 | 3.45 | (2.72, 4.36) | 0.000 |
| **RA** |  |  |  |
| TyG_Q1 |  |  |  |
| TyG_Q2 | 1.44 | (0.76, 2.71) | 0.260 |
| TyG_Q3 | 1.33 | (0.67, 2.61) | 0.408 |
| TyG_Q4 | 4.17 | (2.17, 7.96) | 0.000 |
| TyG-BMI_Q1 |  |  |  |
| TyG-BMI_Q2 | 0.89 | (0.49, 1.58) | 0.685 |
| TyG-BMI_Q3 | 0.44 | (0.22, 0.86) | 0.018 |
| TyG-BMI_Q4 | 1.83 | (1.02, 3.28) | 0.042 |
| **Gout** |  |  |  |
| TyG_Q1 |  |  |  |
| TyG_Q2 | 1.48 | (0.81, 2.66) | 0.195 |
| TyG_Q3 | 1.58 | (0.88, 2.81) | 0.125 |
| TyG_Q4 | 2.68 | (1.51, 4.75) | 0.001 |
| TyG-BMI_Q1 |  |  |  |
| TyG-BMI_Q2 | 1.55 | (0.78, 3.06) | 0.208 |
| TyG-BMI_Q3 | 3.80 | (2.00, 7.17) | 0.000 |
| TyG-BMI_Q4 | 7.34 | (3.86, 13.94) | 0.000 |
| Abbreviation: KOA, knee osteoarthritis. | | | |
